# Optimal foraging by a large ungulate in an extreme environment: Wild mountain reindeer select snow‐free feeding habitats in winter

**DOI:** 10.1002/ece3.7843

**Published:** 2021-07-05

**Authors:** Lena Romtveit, Olav Strand, Anders Mossing, Leif Kastdalen, Arne W. Hjeltnes, Dag K. Bjerketvedt, Arvid Odland, Jan Heggenes

**Affiliations:** ^1^ Norwegian Wild Reindeer Centre Skinnarbu Rjukan Norway; ^2^ Norwegian Institute for Nature Research Trondheim Norway; ^3^ Department of Natural Sciences and Environmental Health University of Southeast Norway Bø i Telemark Norway

**Keywords:** alpine habitat, optimal foraging, snow‐free habitats, wild reindeer, winter

## Abstract

Optimal foraging models predict that individual animals will optimize net energy gain by intensifying forage activity and/or reducing forage energy cost. Then, the free distribution model predicts an animal's distribution in a patchy landscape will match the distribution of the resources. If not modified by other factors, such patterns may be expected to be particularly explicit in variable and extreme, forage‐limited, and patchy environments, notably alpine and Arctic environments during winter. The large ungulate wild mountain reindeer (*Rangifer tarandus tarandus*) surviving in such environments is used as a model during the forage‐limited winter season. The largest wild reindeer area in Western Europe (Hardangervidda, 8130 km^2^) is actively managed to sustain 10,000–12,000 wild reindeer. Since 2001, 104 different individuals have been GPS‐tracked at 3‐hr intervals. In winter, mountain reindeer may either choose to seek out and forage in patchy snow‐free habitats, typically on top of wind‐blown ridges, or use energy‐demanding digging through the snow to reach ground forage (cratering). We use late April satellite data from Landsat 5 and 8 (30 × 30 m), airborne laser scanning subsampling (processed to 1 × 1 m grid), and topographic information (1 m resolution) derived from digital aerial photographs (0.25 × 0.25 m resolution) to delineate snow‐free patches, constituting less than 694 km^2^. By overlaying recorded wild reindeer GPS positions winters 2001–2017 (188,942 positions), we document a strong positive selection for snow‐free patches, which were used about four times more frequently than expected from a “random walk” model. On a daily basis, the preference for snow‐free areas was slightly stronger in the evenings. In the sustainable management of wild mountain reindeer, the area of snow‐free patches is an important predictor of winter forage availability and important winter source areas. It may be derived from remote sensing data.

## Graphical abstract

Remote sensing data were used to delineate typically snow‐free or near snow‐free patches within a 8,600 km^2^ alpine mountain reindeer area. Comparison with wild reindeer GPS positions across winters 2001–2017 documented a strong positive selection for snow‐free patches (four times more use). In the sustainable management of wild mountain reindeer, area of (near) snow‐free snow‐free patches is an important predictor of winter carrying capacity.

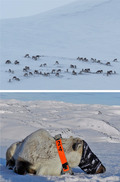

## 1 INTRODUCTION

In a world of limited foraging resources, animals may enhance their fitness by intensified foraging to increase energy gain and/or reduce foraging cost. Optimal foraging theory, with focus on patchy environments, predicts that foraging behaviors optimize net energy gain, the resulting gain per unit food exceeding the loss (MacArthur & Pianka, 1966; Perry & Pianka, 1997; Pyke, 1984). As a corollary, the ideal free distribution (IFD) model predicts that the distribution of an organism between resource sites should match the distribution of the resources (Fretwell & Lucas, 1970; Křivan et al., 2008; Sutherland, 1983). The distribution of organisms may however be less extreme than the distribution of the resource, that is, modified by organism discrimination constraints, competitive interactions, competitive asymmetries, and travel between sites (Abrahams, 1986; Calsbeek & Sinervo, 2002; Kennedy & Gray, 1993) or simply haphazard choices, and shift toward an ideal despotic distribution. The presence of at least elements of these theories in animal distributions and behaviors have been demonstrated across a variety of animal groups (DeAngelis, 2018; Perry & Pianka, 1997; Pyke, 1984), and as may be expected, the theories have diversified over time with empirical data from diverse groups and environments (Arditi & Dacorogna, 1988; Robinson & Wilson, 1998; Ward, 1992). Fluctuating and extreme environments present particular challenges: Environmental persistence should profoundly influence behaviors when animals have to deal with such variability and maximize their survival (Higginson et al., 2012).

High‐latitude alpine and Arctic ecosystems are highly variable and harsh environments, across seasons and years, with low productivity and patchy foraging habitats, particularly during the extremely long winter season. A surprisingly large animal that survives in these environments is the cold‐adapted reindeer *Rangifer tarandus* L. (caribou in North America), the most widely distributed mammalian herbivore in these northern ecosystems (Skogland, 1983, 1984). Reindeer live in herds, are almost constantly in motion, graze extensively, and exhibit some of the longest ungulate migrations known, because of the typically low forage production and patchy distribution of high‐quality vegetation resources in high‐altitude environments (Falldorf et al., 2014; Hansen et al., 2009; Johnson et al., 2001). Although summer conditions and opportunities for fat storage may set preconditions, it is the extreme winter which is the typically critical survival period, when foraging resources are patchy and limited, depending on snow and ice cover, in particular for the mountain reindeer (*Rangifer tarandus tarandus* L.). Reindeer movements tend to be less and more regular than during other seasons (Strand et al., 2006), and potential diel rhythmicity may be attenuated (Arnold et al., 2018; Loe et al., 2007). Many reindeer populations live in environments where they need to take the cost of digging in situ feeding craters in the snow (Fancy & White, 1985), typically to a depth 50–70 cm or less, depending on snow conditions (Fancy & White, 1985; LaPerriere & Lent, 1977; Skogland, 1978), to reach plant cover (Ferguson et al., 2001; Hansen et al., 2010; Kumpula et al., 2004). Reindeer try to reduce energetic cost when digging for food in winter by avoiding areas with deep or hard snow (Skogland, 1984). Here, we explore if reindeer may extend this behavior into an alternative foraging strategy. In particular in exposed mountain areas, reindeer may choose to travel, taking the relatively low energetic cost of locomotion, depending on snow conditions (Fancy & White, 1987), and preferably forage on exposed, wind‐blown, snow‐free habitats on elevated ridges. Such habitats favor slow‐growing ground lichen in particular (Odland & Munkejord, 2008b; Odland et al., 2018). Therefore, we hypothesize that mountain reindeer in winter exhibit a strong preference for snow‐free areas, clustered within relatively short distances. If so, such areas may be an important predictor of reindeer area use and carrying capacity in winter. Snow‐free areas may be identified from remote sensing data (e. g., Härer et al., 2018) and may constitute important source habitats to be sustained in reindeer management.

Here, we combine accurate GPS positioning with remote sensing techniques to test the hypothesis that wild reindeer will, for energetic reasons, select to forage on snow‐free areas and according to the IFD theory be more frequently associated with snow‐free patches than alternative areas.

## 2 METHODS AND MATERIALS

### 2.1 Study area

**FIGURE 1:**
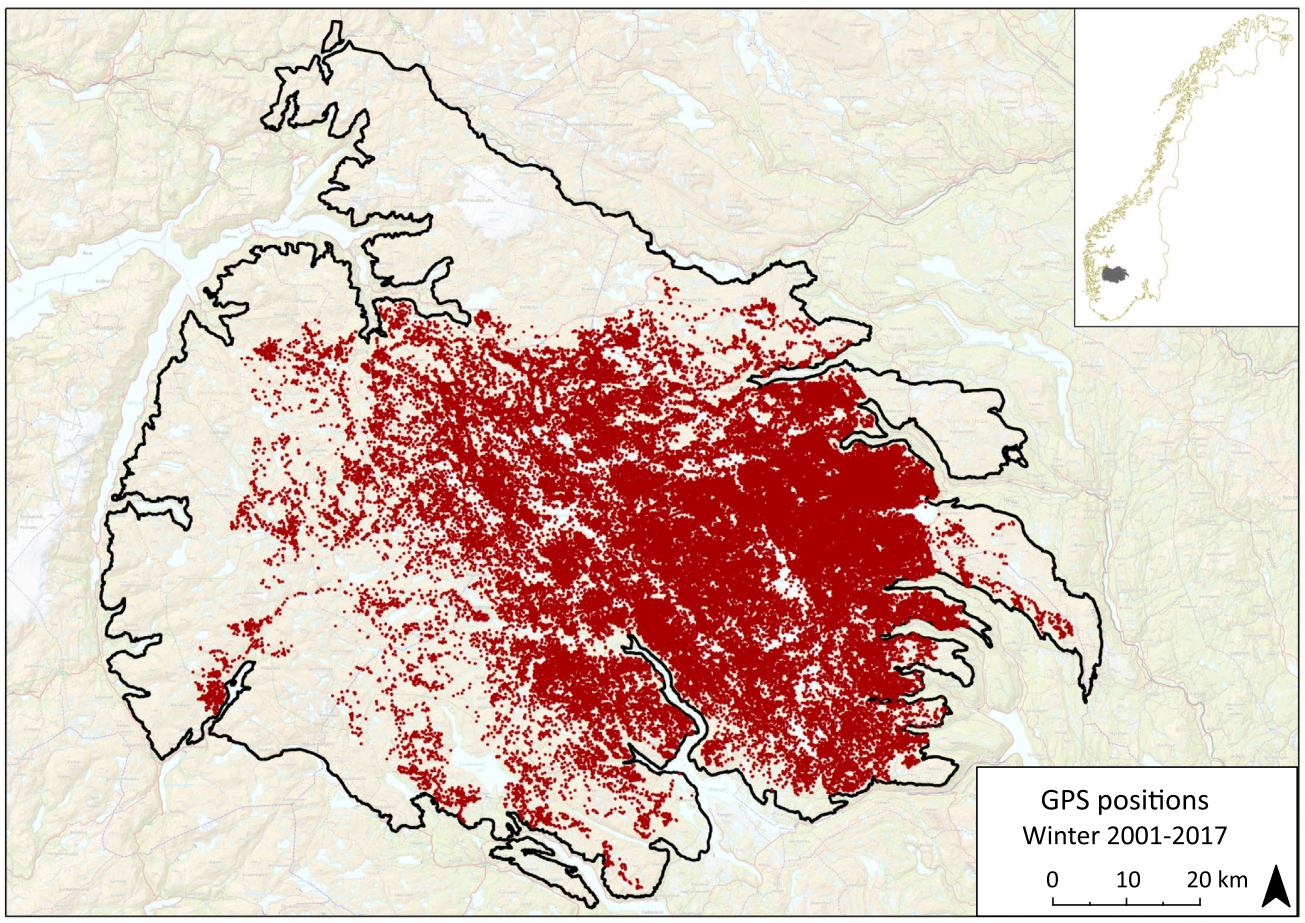
Spatial distribution of GPS mountain reindeer winter (December‐April) positions (*n* = 188,942) 2001–2017 on Hardangervidda, south‐central Norway, with a west–east climate gradient from oceanic to continental, that is, high to low precipitation. Solid line delineate the wild reindeer area (8,130 km^2^). Inset: location in Norway

The alpine Hardangervidda (about 10,000 km^2^, mostly 1,100–1,300 meter above sea level) in southern Norway is the largest mountain plateau in Europe and harbors the last remaining wild mountain reindeer in Western Europe (Figure 1) (Gaare & Skogland, 1975; Østbye et al., 1975; Wielgolasky & Kjelvik, 1973). Although other wild reindeer populations are fragmented because of anthropogenic activities, the Hardangervidda population still maintains a near‐natural seasonal migration pattern, largely determined by foraging resource distribution (Falldorf, 2013; Nilsen & Strand, 2017; Strand, 2009; Strand et al., 2006). A dominant west (oceanic)–east (continental) climate gradient, geology, and topography combine to generate strong vegetation mosaic patterns. By altitude, the low alpine zone stretches from the tree line (ca. 1,100 m.a.s.l.) and about 300 m upwards. Areas higher than ca. 1,400 m.a.s.l. belong to the mid‐alpine zone (Moen, 1999). The vegetation is dominated by perennial species with less than 10% of therophytes. Of the approximately 130 vascular species found in the low‐medium alpine areas, hemicryptophytes make up more than 50%. Total live biomass of vascular plants and cryptogams may be 300–700 g m^−2^. Although Hardangervidda is classified as tundra, there is no permafrost, one reason being the stable and relatively thick snow cover during the long winter (Østbye et al., 1975). Typically, maximum snow depth (expressed as mm water equivalent) may decrease from more than 2,000 mm in the west to 100–250 mm in the east (http://www.senorge.no/index.html?p=senorgeny&st=snow), but with substantial variation, depending mainly on topography and wind. Snow is blown away from exposed sites and redistributed to terrain depressions and lee‐sides. The relatively stable prevailing wind directions generate larger‐scale snow distribution patterns (Sturm & Wagner, 2010), although snow precipitation and thickness may vary from year to year. Together with low temperatures, this is the main factor dictating development of vegetation communities in alpine areas. On oligotrophic, dry, wind‐blown ridges maximum snow cover is often less than 50 cm. Such areas are typically dominated by lichens (Dahl, 1956) and known as lichen heaths. Lichen‐dominated heaths mainly develop on soil more than ca. 15 cm deep and with soil frost sum higher than 200, which is typical for snow‐free patches on ridges (Odland et al., 2018). The average lichen biomass of such sites may vary from 200–800 gm^−2^ or more in ungrazed areas to below 100 gm^−2^ or less in grazed areas (Odland et al., 2014). Lichen constitute the most important forage for wild mountain reindeer during winter on Hardangervidda (Falldorf, 2013; Gaare & Skogland, 1975; Skogland, 1984). With declining altitude, vegetation grades to scattered graminoids and dwarf shrubs and then to areas with more, higher vegetation, which parallels the snow‐layer duration gradient (Odland & Munkejord, 2008a; Odland et al., 2014).

Natural predators have been extinct from the area since late 1800s, with the exception of golden eagle (*Aquila chrysaetos*) and an occasional wolverine (*Gulo gulo*), and the population is regulated by hunting, with historically varying population densities (Bjerketvedt et al., 2014). In winter, the reindeer migrate to the central and eastern areas with less snow precipitation (Figure 1) (Kastdalen, 2011; Strand et al., 2006). However, two tongues of land, in total area 656 km^2^ in the northern and eastern parts of Hardangervidda (Figure 1), are not or rarely used in winter by reindeer, due to the presence of roads and cabins which act as semi‐barriers. These areas were excluded from the analyses. Since 2001, reindeer on Hardangervidda have been fitted with GPS transmitters, currently providing one of the largest datasets of ungulate positioning (Strand et al., 2015).

### 2.2 Reindeer positioning

GPS tracking of reindeer on the Hardangervidda was conducted 2001‐2017, with 8–10 females being marked annually (no marking in 2006) (Strand et al., 2006, 2015). Permits for capture and sampling and including ethical considerations were acquired from the Norwegian Food Safety Authority (FOTS: ID 15116, license # 19/20935). Females are chosen because their behavior is more representative of the herd and translates more directly into population demography (Strand, 2009; Strand et al., 2015). Using a helicopter, groups of reindeer were identified and an arbitrarily selected female was immobilized with medetomidin/ketamine 12/120 mg 3 ml arrows fired from a Danarms℗ CO^2^ air rifle into the animal's thigh. Blood, hair, and tissue samples were collected and a radio/GPS unit attached to a drop‐off collar was fitted. Temperature, pulse, respiration, and blood O_2_ were monitored during the operation. Constriction was avoided by fitting collars with four (male) finger‐widths of slack and the presence of a cotton weak zone. Although designed to drop off in response to a radio signal, most collars dropped off spontaneously within two years. A position signal was sent every 3 hr for up to 3 years (the battery life). The position time series used here (2001–2017) consisted of 188 942 winter positions (December‐April) out of a total of 609 350 positions throughout the years from 104 reindeer (Figure 1) (for details see Strand et al., 2006, 2011, 2015). Frequent signals facilitated analysis of potential diurnal patterns in spatial positions.

### 2.3 Analyses

In an use‐versus‐available study design (Manly et al., 2002), we explored if availability of snow‐free areas influenced reindeer position choice.

#### 2.3.1 Geomatics analyses

**FIGURE 2:**
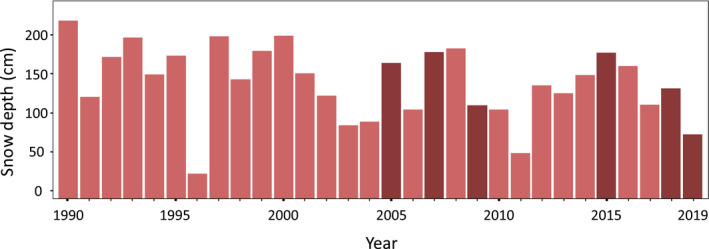
Snow depths (cm) on central Hardangervidda 1980–2019. The 6 years with dark color provided cloud‐free (<4%) Landsat satellite images for analysis of snow‐free patches

Snow‐free patches were first identified and delineated by using satellite data from Landsat 5 and 8 with 30 × 30 m resolution. Within the time window late April, when snow cover typically is most extensive (Kohler et al., 2006; Lawrence & Slater, 2010), we found six acquisitions covering the whole study area and with close to cloud‐free conditions (0%–4% clouds). Based on daily estimates of snow depth in a 1 km grid (http://www.senorge.no/aboutXgeo.html) (Saloranta, 2012; Strand et al., 2006), we estimated annual mean snow depths (at April 25th) for the central study area (Figure 2). Two years (2005, 2018) had snow depths close to normal, two years less than normal (2009, 2019), and two years more than normal (2007, 2015) (Figure 2). On average, they likely represent the typical snow situation, with the six used years having the same mean snow depth as the 30 years average, that is, 131 cm on 25 April.

**FIGURE 3:**
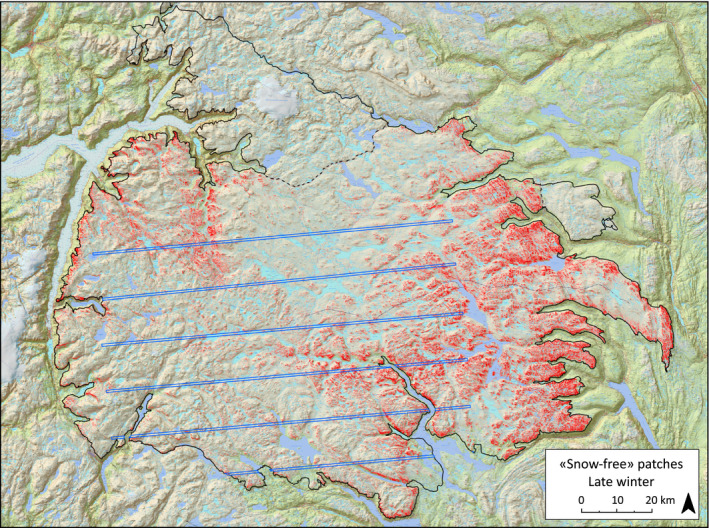
Spatial distribution of delineated snow‐free patches (red color, defined as 50% or more snow‐free areas within a pixel, *n* = 771,180) on Hardangervidda, south‐central Norway, based on Landsat satellite images (30 × 30 m resolution) and the Normalized‐Difference Snow Index and Topographic Position Index, modeled with data from ALS subsampling (1 × 1 m grid within the blue 500 m wide transects). Dotted lines indicate two areas not used by reindeer and not included in the analyses. Solid line delineate the wild reindeer area

Initially, we used the snow mask produced by the Unites States Geological Survey (USGS) and included in the Landsat surface reflectance products, to represent snow‐free areas within the study area. For control, outputs were compared with fieldwork and independent data provided by high‐resolution airborne laser scanning (ALS) subsampling within the study area (processed to 1 × 1 m grid, see below), and visual inspection of plant communities derived from 4‐channel, 25 cm resolution aerial photographs (community structure derived from an adjacent southern mountain area (Figures 1, 3; Brattefjell‐Vindeggen) (Hjeltnes et al., 2017; Lunetta & Lyon, 2004; Paul et al., 2016)). ALS data were from a subsample consisting of six 500 m wide, uniformly spaced, east–west transects (Figure 3), which were flown in September 2008 (ground level) and April (snow surface) 2008 and 2009 (Melvold & Skaugen, 2017).

The global USGS snow mask, based on a threshold value from spectral bands, did not identify snow‐free areas sufficiently well for our purpose, a result also seen for other European areas (Härer et al., 2018). Therefore, we developed a local snow‐fraction model based on the 1 × 1 m grid ALS data with each pixel classified as snow‐free or snow‐covered and align these with the corresponding 30 m Landsat grid to derive the aggregate snow‐fraction for each 30 × 30 m pixel within the subsampled transects, and ranging from 0, no snow cover 1 × 1 m ALS pixel within the 30 m Landsat unit, to 1, all ALS pixels snow‐covered. The ALS intensity image from the 2009 data (acquired April 21–24, 2008 acquisitions were more spread out in time, April 3–21, and with more snow than normal, Figure 2), indicated a snow depth threshold value of 35 cm or less for classifying a 1 × 1 m ALS pixel as snow‐free, as such pixels were identified as “black,” that is, snow‐free. To predict snow cover in these subsampled 30 × 30 m Landsat pixels from Landsat data, we used the mean value of the Normalized‐Difference Snow Index (NDSI) across the six available Landsat scenes. The mean value showed a stronger correlation to the ALS‐based snow‐fraction estimates than any of the single scenes (2009 data; Pearson correlation coefficient *R* = −0.72). From the six Landsat scenes combined with a 1 m digital elevation model (DEM) (Hingee et al., 2016), we also derived the geomorphometric indices Topographic Position Index (TPI) (Newman et al., 2018; window size 30 m), slope, and hill‐shade (depending on illumination angle and direction), averaged to fit the Landsat 30 meter grid size. However, only TPI was kept as the second predictor to model the response snow cover. TPI had the higher correlation to the response (*R* = −0.41). Slope and hill‐shade did not improve the model and were dropped from further analyses. Based on the calibrated NDSI and TPI predictors, we modeled snow cover for all Landsat pixels across the study area. Because of a curvilinear relationship for both the NDSI and TPI predictors with the ALS‐estimated snow‐free pixel fraction, and a threshold effect for the TPI, models were developed using flexible algorithms like generalized additive models (GAM) (Wood, 2018) and the machine learning algorithms Cubist (Kuhn et al., 2012), Xtreme Gradient boosting (Chen et al., 2015), and Random forest (Breiman, 2001), included in the R package Caret (Kuhn & Johnson, 2013). To avoid using reference data highly skewed toward snow‐covered areas, random selection was used to extract approximately the same number of data points for each group of 10% percent increase in fraction of snow cover. Model performances were evaluated with five‐fold cross‐validation repeated five times (25 resamples), and for all models, the *R*² values were between 65.5% and 66.6%. To increase model robustness, we averaged results across the two best models (Cubist and Gradient). The snow‐fraction model also facilitated alternative scenarios with increasingly strict threshold values for classifying an area as “snow‐free,” and for relevant comparisons, we used 80%, 50%, and 30% coverage of 1 × 1 m snow‐free ALS pixels within a Landsat 30 m grid cell to consider it as snow‐free.

Manual control with a Landsat‐based vegetation map (Mossing et al., 2009) indicated that a few, large, low‐altitude patches along the outer south‐east margins of the reindeer area, and not, or rarely, used by reindeer (Figure 1), were misclassified as snow‐free because of the light signal from the stems of the dense tree line birch forest. These patches, constituting 6.9% of the snow‐free patch area, were excluded from analyses.

### 2.4 Statistical analyses

Spatial data for available habitat, that is, snow‐free areas and habitat use by reindeer, that is, GPS positions, were combined and analyzed using the software ArcPro 2.5 (ESRI, 2011) for spatial overlay data and R v. 3.5 (R Core Team, 2014) for further statistical analyses, in particular the “raster” package (Hijmans, 2020).). The map of snow‐free areas was overlaid with reindeer GPS positions during winter (December–April) and analyzed for two scenarios with contrasting spatial resolution. First, snow‐free areas, as estimated from Landsat data, were overlaid with all GPS positions in the reindeer area. Second, from the ALS subsample within transects we explored potential daily differences in habitat use, comparing day (sunrise +1 hr. to sunset −1 hr.), evening (sunset ±1 hr.), night (sunset +1hr. to sunrise −1 hr.), and morning (sunrise ±1 hr.) positions. We also developed Resource Selection Probability Functions (RSPF) sensu Lele (2009) and Lele et al. (2013) for more detailed analysis of biologically plausible predictors of reindeer positions, using the R package ResourceSelection (logistic model, cloglog link) (Lele et al., 2019). In addition to estimating the binary variable snow‐free areas (or not), the ALS data also facilitated estimation of additional, continuous predictor variables. Plausible predictors explored were snow depth (SNOW; cm), distance to snow‐free areas (DIST; within ALS transects: m to nearest median‐sized snow‐free area (1,800 m^2^), else: m to 30 × 30 m pixel more than 30 % snow‐free as estimated by Landsat data), lichen cover (LICHEN; %, 30 × 30 m pixel) (Mossing et al., 2009), elevation (DEM and DEM^2^, quadratic term to reflect that reindeer may prefer areas located within the middle of the used elevation range), TPI (30 × 30 m pixel, calculated from a DEM with 1 m resolution and 30 m window size) and X‐coordinate (XCOORD; proxy for east–west climate gradient). Predictors were standardized before analyses, by subtracting the mean and dividing by the standard deviation (Schielzeth, 2010). Availability was modeled by 34,202 randomly selected 1 × 1 m ALS pixels within transects (all pixels numbered, random numbers selected). Reindeer had used 3 653 such pixels. Because GPS positioning accuracy may deviate by a few meters, but, if present, no more than 5–6 m (Falldorf, 2013; Jung et al., 2018), we ran analyses with a 5 m buffer zone for each GPS position, selecting the 1 × 1 m pixel with the lowest snow depth within that buffer zone as animal position. Akaike information criterion (AIC) was used to compare the relative fit of the different models tested (Akaike, 1974; Burnham & Anderson, 2002), exercising caution in interpretation if models included additional parameters, but were within 2 AIC units of the top‐ranking model (Arnold, 2010). Predictors used in the analysis were not strongly correlated (*R* < 0.38 and variance inflation factor < 2) (Harrison et al., 2018).

## 3 RESULTS

The total area of the Hardangervidda reindeer area considered available to reindeer is 7474 km^2^, excluding one northern and one eastern land tongue not, or rarely, used by reindeer (Figure 1).

**FIGURE 4:**
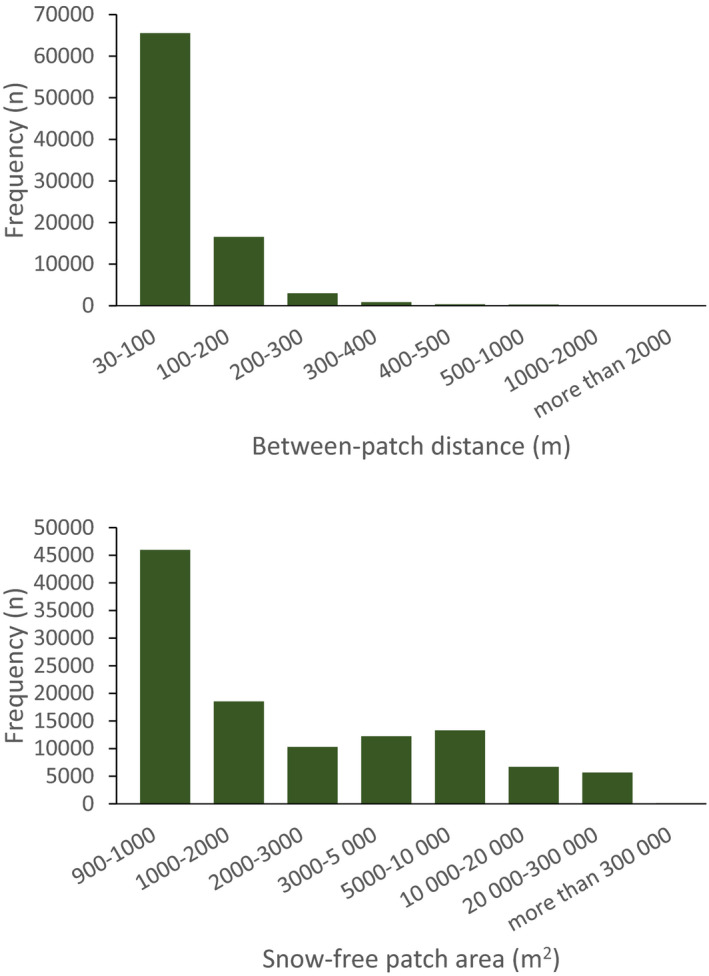
Inter‐patch Euclidean distances (top), and size distribution of delineated snow‐free patches (bottom) on Hardangervidda, for 50 % or more snow‐free areas within a Landsat satellite image pixel (30 m × 30 m resolution), controlled by ALS subsampling (1 × 1 m grid) and digital aerial photographs (0.25 × 0.25 m resolution)

**FIGURE 5:**
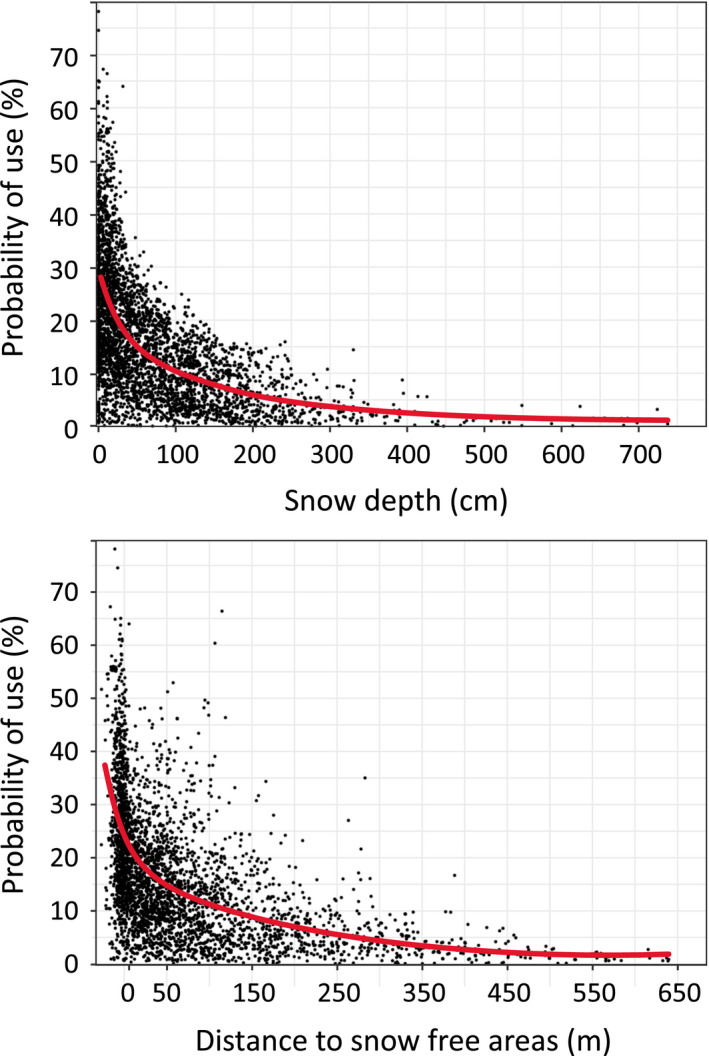
Predicted probabilities by the best Resource Selection Probability Function (relative) for no or low‐snow depth (top) and distance to snow‐free areas (bottom) for GPS‐positioned wild reindeer (*n* = 3,653) in subsampled ALS transects, on Hardangervidda, south‐central Norway. Line based on snow depth as mean of 2008 and 2009

Snow‐free areas for the alternative threshold values of 80 %, 50 %, and 30 % (snow‐free fraction per 30 × 30 m Landsat pixel), were estimated to cover, respectively 107 km² (1.4 %), 694 km² (9.4 %), and 1,344 km² (18.0 %) (Figures 2, 3) of the total area in a winter with “average” snow conditions (Figure 2). The size distribution of these snow‐free areas (Figures 3, 4) for the two stricter thresholds (80 %: *n* = 37,344; 50 %: *n* = 110,113) was skewed with a strong dominance of small, clustered patches (median = 1,800 m^2^) (Figure 4). Between‐patch distance was typically short (median = 90 m, 64 % ≤ 100 m) (Figure 4). On a large spatial scale, the reindeer winter GPS positions generally reflected the overall distribution of snow‐free or near snow‐free areas (Figures 1, 3, 5). Across the winters, mountain reindeer exhibit a strong preference for snow‐free areas. In total, 56,378 reindeer positions were on snow‐free areas as estimated by remote sensing, and with a 50 % threshold value, that is, (56,378/188,942)*100 = 29.8 %, which is three times more than expected if reindeer used winter areas proportionately (17,383 positions expected; Odds Ratio = 4.20 (95% CI 4.1206–4.2755, *p* < .0001)). For the stricter analysis based on an 80 % threshold value, the preference for snow‐free patches was even stronger. An estimated 11,597 reindeer positions were on snow‐free patches, which is more than four times more than expected if reindeer used these winter areas proportionately (2,645 positions expected; Odds Ratio = 4.61 (95% CI 4.4132–4.8069, *p* < .0001)). Correspondingly, for the more relaxed 30 % threshold value, the preference was not as strong, but still clearly significant (87,340 reindeer positions, that is, (87,340/188,942)*100 = 46.2 %, and 2.6 times more than expected by proportionate use (33,976 positions expected; Odds Ratio = 3.92 (95% CI 3.8628–3.9791, *p* < .0001)).

The detailed ALS and RSPF analyses corroborated this pattern. Within the six 500 m wide ALS transects, GPS‐positioned wild mountain reindeer exhibited a strong probability of selecting no or low‐snow depth areas (Figure 5), with a rapidly increasing preference for snow depths less than 1 m, and shorter distances to snow‐free areas. On a finer temporal scale, the preference for snow‐free patches was somewhat stronger in the evenings (sunset ±1 hr.), particularly for the 80 % threshold (70 % more observations than expected; 50 % threshold: 26 % more observations; 30 % threshold: 20 % more observations). No clear pattern emerged for other times of day or night. In all tested Resource Selection Probability Function (RSPF) models (Table 1), snow depth and closeness to snow‐free areas were strongly significant predictors (*p* < .0001). The best and full RSPF model (Table 1), included distance to snow‐free area (coefficient = −0.46 (17 %), SE = 0.040, *p* < .0001) and snow depth (coefficient = −0.35 (13 %), SE = 0.041, *p* < .0001), together with X‐coordinate (coefficient = 0.77 (29 %), SE = 0.0030, *p* < .0001), elevation^2^ (coefficient = −0.31 (11 %), SE = 0.025, *p* < .0001), and elevation (coefficient = 0.27 (10 %), SE = 0.032, *p* < .0001), TPI (coefficient = 0.18 (7 %), SE = 0.024, *p* < .0001) lichen cover (coefficient = 0.11 (4 %), SE = 0.026, *p* = .0001), and interaction snow depth*lichen (coefficient = −0.23 (9 %), SE = 0.034, *p* < .0001). Still, there was additional variation not covered by this best model (Hosmer‐Lemeshow goodness‐of‐fit; X^2^ = 69.01., *df* = 8, *p* < .0001).

**TABLE 1:** The tested RSPF models ranked by AIC

Rank	Predictors full model—removed	AIC	∆AIC
1	DIST + SNOW + XCOORD + DEM2 + DEM + TPI + LICHEN + SNOW:LICHEN	72,103.0	0
2	‐ TPI	72,162.9	47.7
3	‐ SNOW	72,249.2	146.2
4	‐ LICHEN	72,202.5	200.5
5	‐ DIST	72,420.6	317.6
6	‐ DEM/DEM2	72,464.5	361.5
7	‐ XCOORD	73,274.1	1,171.1

Note

∆AIC = the difference between AIC for the model of concern compared to the best‐ranked model.

## 4 DISCUSSION

### 4.1 Reindeer foraged on snow‐free areas

The results clearly indicate that, where the choice is available, wild mountain reindeer exhibit a preference for snow‐free or snow‐poor habitat areas in winter. For the large dataset used here, the reindeer distributed themselves in much more close association with snow‐free patches that would be expected from a “random walk” model, that is, use in proportion to availability. In wintertime, wild reindeer individuals on Hardangervidda spend most of their time foraging (Skogland, 1984). Reindeer may seek out snow‐free forage patches or alternatively dig through the snow to reach forage (“cratering”), which presumably has a substantial energetic cost (Fancy & White, 1985). Optimal foraging favors the strategy alternative of foraging on snow‐free patches, given relatively low travel distances between patches, as documented here, and the high energetic efficiency of locomotion in reindeer (Fancy & White, 1987). Moreover, patches were many and small, and mainly located in areas with overall low‐snow depths, which also offer opportunities for “cratering” at the edges of and between patches. However, “cratering” may be less energetically efficient also because of the limited potential net energy gain associated with the small vegetation area uncovered by cratering, but depending on snow conditions (Helle, 1984; Kumpula et al., 2004; LaPerriere & Lent, 1977; Pruitt, 1959). When given a choice, snow‐free patches offer much larger and easily available vegetation. Even though lichen biomass in snow‐covered areas is typically greater—up to 800 g/m^2^ or more—(Kumpula et al., 2011; Odland et al., 2014), while that of exposed, snow‐free patches may be down to 100 g/m^2^ or less if grazed, this may be offset by the limited ground area uncovered by cratering.

### 4.2 Resource selection predictors

The best RSPF model was obtained including the predictors snow depth, distance to snow‐free areas, topographic position at a local scale (30 m window), elevation, X‐coordinate, and lichen cover. This lends support to our main result, the preference for snow‐free or snow‐poor habitat areas in winter. Indeed, elevated, exposed, wind‐blown ridges provide habitats that favor slow‐growing ground lichen (Odland & Munkejord, 2008b; Odland et al., 2018). Such habitats are found more frequently going east on Hardangervidda, because of the climate gradient (Figures 1, 3) (Gaare & Skogland, 1975; Moe, 1995; Skartveit et al., 1975), which the X‐coordinate is a proxy for. The drier climate to the east likely also results in less packed snow and ice/crust layers. We are not aware of similar studies of the effect of snow‐free patches on reindeer foraging in Europe, nor on barren‐ground caribou in North America. Notably, also barren‐ground caribou show a preponderance of lichen in their winter diet (Åhman et al., 2018; Joly & Cameron, 2018; Joly et al., 2015; Thomas & Hervieux, 1986), and forage preferentially where snow cover is softer, lighter, and thinner (e.g., less than 50–60 cm) (Miller, 2000; Pruitt, 1959).

Reindeer are seasonal animals accumulating energy reserves in summer, and living mainly off these reserves in winter (e. g., Åhman et al., 2018). Lichen, which was a significant predictor in the best RSPF model, is used preferentially as winter maintenance forage. Although reindeer seem to favor the carbohydrate‐rich lichen (Danell et al., 1994; Hansen et al., 2010; Vistnes & Nellemann, 2008), which also happens to be relatively easy to differentiate and monitor by remote sensing (Kastdalen, 2011; Mossing et al., 2009), its importance should not be overstressed. When available, free‐living reindeer may eat a mixture of lichens, mosses, and vascular plants such as shrubs and graminoids in winter (Mathiesen et al., 2000; Skogland, 1984; Storeheier et al., 2002), and select for medium to high‐quality forage rather than low‐digestible high‐biomass forage (Hansen et al., 2010). Warenberg (1982) found that many plants growing in snow‐poor habitats have green buds in winter. Chemical analysis indicates relatively high contents of crude protein and minerals in these plants in winter (Storeheier et al., 2002), which would be of great benefit to reindeer. Availability of snow‐free areas may be more important than availability of lichen.

### 4.3 Identification of snow‐free areas by remote sensing techniques

From the satellite images, we estimated area of snow‐free patches to about 1.4 resp. 9.3 % of total area. These estimates are comparable with previous estimates, based on other methods (Bjerketvedt et al., 2012; Hesjedal, 1975; Jordhøy & Strand, 2009). These previous estimates are similar to our higher (50 % threshold value) estimate, but markedly higher than our stricter 80 % threshold value. Our estimate did not include the now unused northern and the eastern land tongues, the latter which, in particular, holds substantial potential winter forage areas (Jordhøy & Strand, 2009). Many of the smaller snow‐free patches may consist of exposed rocks, less suitable habitat for winter forage. Lichen‐dominated heaths are more often found on the somewhat larger, exposed ridges (Hesjedal, 1975; Odland et al., 2018). These areas would have no or little snow cover across a wide range of snow‐fall conditions and would thus be consistently important source forage habitats. The reduced area estimates and stronger preference ratios with increasingly strict model thresholds likely reflect this ecological feature. Exposed ridges may be particularly important in winters with much snow. Estimates of snow‐free patches, particularly for the more relaxed model assumptions, will be affected by the annually varying snow precipitation, criteria for when a pixel should be classified as snow‐free, and at which time in winter/spring available Landsat scenes are recorded, the transition between snow accumulation and snow melt being very dynamic.

## 5 CONCLUSION

Selection for snow‐free habitats by wild reindeer during the commonly extreme winter conditions appears to conform well with ecological theory, that is, optimal net energy gain foraging and ideal free distribution (e. g. Křivan et al., 2008; Perry & Pianka, 1997; Pyke, 1984). Such behaviors may be modified by risk (Bernstein et al., 1988; Lima, 2002; Moody et al., 1996), but, since there is no predation of significance, risk is virtually absent for wild reindeer in the investigated area. Snow‐free and low‐snow depth areas may be important predictors of available forage that should be considered in sustainable mountain reindeer management. Such areas may be source habitats during winter that cannot easily be replaced and are therefore important to protect from anthropogenic disturbance, land use, and development. Conveniently, snow‐free and low‐snow depth areas may be identified using remote sensing data.

## CONFLICT OF INTEREST

None.

## AUTHOR CONTRIBUTION

**Lena Romtveit:** Data curation (equal); Funding acquisition (equal); Investigation (lead); Resources (equal); Software (equal); Validation (equal); Writing‐original draft (equal). **Olav Strand:** Conceptualization (equal); Data curation (lead); Funding acquisition (equal); Investigation (equal); Resources (equal). **Anders Mossing:** Conceptualization (equal); Data curation (equal); Formal analysis (equal); Funding acquisition (equal); Resources (equal); Visualization (equal). **Arne William Hjeltnes:** Data curation (equal); Formal analysis (equal); Investigation (equal); Methodology (equal); Resources (equal); Software (equal); Validation (equal); Visualization (equal). **Leif Kastdalen:** Data curation (equal); Formal analysis (lead); Investigation (equal); Methodology (equal); Resources (equal); Software (lead); Validation (lead); Visualization (equal). **Dag Kjartan Bjerketvedt:** Conceptualization (equal); Formal analysis (equal); Funding acquisition (equal); Investigation (equal); Resources (equal); Supervision (equal); Validation (equal). **Arvid Odland:** Conceptualization (equal); Funding acquisition (equal); Investigation (equal); Resources (equal); Supervision (equal). **Jan Heggenes:** Conceptualization (equal); Data curation (equal); Formal analysis (equal); Funding acquisition (lead); Methodology (equal); Resources (equal); Supervision (equal); Writing‐original draft (lead); Writing‐review & editing (lead).

## Data Availability

‐ GPS positions may be viewed at: https://www.dyreposisjoner.no/Account/Login?ReturnUrl=%2F Developed and maintained by Norwegian Institute of Nature Research (NINA).
‐ Satellite images downloaded from: https://earthexplorer.usgs.gov/ https://scihub.copernicus.eu/dhus/#/home
‐ Aerial photographs downloaded from: https://www.norgeibilder.no/ and snow data may be viewed at: http://www.senorge.no/index.html?p=senorgeny&st=snow Developed and maintained by the Norwegian Mapping Authority (SK), Norwegian Institute of Bioeconomic Research (NIBIO) and the State Highways Authority. All data are also archived and publicly available at the Norwegian Wild Reindeer Centre, Skinnarbu, 3660 Rjukan, Norway. Data also in public repository Dryad https://doi.org/10.5061/dryad.d2547d831.
